# Supplementary material for: Hydrophobically Modified Isosorbide Dimethacrylates as a Bisphenol-A (BPA)-Free Dental Filling Material
Source: Materials (Basel). 2021 Apr 22;14(9):2139. doi: 10.3390/ma14092139 (PMC8122847; doi:10.3390/ma14092139)
Supplement: Supplementary file 1 [file materials-14-02139-s001.zip › materials-1172283-supplementary.pdf]

materials

MDPI

Article

Hydrophobically Modified Isosorbide Dimethacrylates as a Bisphenol-A (BPA)-Free Dental Filling Material

Bilal Marie [1,2], Raymond Clark [2], Tim Gillece [2], Seher Ozkan [2], Michael Jaffe [1] and Nuggehalli M. Ravindra [1,*]

[1] Interdisciplinary Program in Materials science and engineering, New Jersey Institute of Technology, Newark, NJ 07012, USA; bm253@njit.edu (B.M.); jaffe@njit.edu (M.J.)
[2] Ashland specialty ingredients, Bridgewater, NJ 08807, USA; rbclark@ashland.com (R.C.); Tgillece@ashland.com (T.G.); Sozkan@ashland.com (S.O.)
* Correspondence: ravindra@njit.edu

1. Synthesis of ISB4GBMA Intermediates

Step 1. Preparation of Methyl 4-(Allyloxybenzoate)

In a 3-neck Morton round bottom flask equipped with a Teflon paddle stirrer, a condenser, and a nitrogen line, 152 g (1.0 mole) of methyl 4-hydroxybenzoate, 276 g (2.0 mole) of anhydrous potassium carbonate and 600 ml of DMF were charged and dissolved. Dropwise addition of 133 g (1.1 mol) of allyl bromide over one hour was followed and the reaction was run for up to 48 hours. At the end of the reaction, 1,000 ml of DI water was added to dissolve the precipitated potassium chloride salt which is later filtered. The organic material is extracted with 250 ml of methylene chloride and mixed with anhydrous magnesium sulfate to remove residual water. The final product is concentrated through a rotary evaporator and distilled under vacuum to afford a clear liquid (92% yield, >98% pure).

^{1}H NMR ($CDCl_3$- δ, ppm): 3.8(3H, $-CH_3$), 4.48 (2H, $-CH_2$), 5.2 (1H, -CH allyl), 5.4-5.5 (1H, -CH allyl), 5.9-6.0 (1H, -CH allyl), 6.8 (2H, -CH aromatic), 7.9 (2H, -CH aromatic)

Step 2: Preparation of Allyl 4-Benzoic Acid

In a 3-neck Morton round bottom flask, equipped with a Teflon paddle stirrer, a condenser, a nitrogen line, a thermocouple, and a heating mantle. 164 gm (0.855 mol) of methyl 4-allyloxy benzoates and 500 ml methanol were added. The mixture was stirred and heated to reflux and 80 g (1.42 mol) of potassium hydroxide in 500 ml DI water were dropwise added over one hour. The reaction was run for up to 48 hours then cooled to room temperature, where 100 ml of 12N hydrochloric acid were added to precipitate the free acid. The product was then filtered and recrystallized in a 750 ml ethanol to yield a white solid (87% yield, >98% pure, mp 163-164°C).

^{1}H NMR (DMSO-d6- δ, ppm): 4.6 (2H, $-CH_2$), 5.3 (1H, -CH allyl), 5.4 (1H, -CH allyl), 7.0 (2H, -CH aromatic), 7.9 (2H, -CH aromatic).

Step 3: Preparation of Isosorbide 2,5-bis(4-Allyloxybenzoic Acid)

In a dry 3-neck Morton round bottom flask, equipped with a Teflon paddle stirrer, a condenser, and a nitrogen line. 73 g (0.50 mole) of isosorbide, 12.6 g (0.1 mol) of 4-dimethylaminopyridine, and 600 ml of DCM were charged and stirred at 0-5°C. 191 g (1.0 mol) of ethylenecarbodimide hydrochloride in 600 ml of DCM were charged dropwise over one hour. The reaction was run for 48 hours at room temperature, and the resulting isourea byproduct was filtered off. The reaction product was washed with 15% hydrochloric acid solution in a separatory funnel to remove any remaining DMAP and the excess acid was later removed with a saturated sodium carbonate solution wash. The organic layer was mixed with anhydrous magnesium sulfate to remove residual water, filtered,

and the product was concentrated in a rotary evaporator and recrystallized with 800 ml ethanol to yield an off-white solid material (65% yield, >89% pure, mp 91-92°C)
^{1}H NMR ($CDCl_3$- δ, ppm): 3.97-4.12 (4H, -CH isosorbide), 4.65 (4H, $-CH_2$), 4.65 (1H, -CH isosorbide), 5.0 (1H, -CH isosorbide), 5.28-5.44 (6H, 2-CH isosorbide, 2-CH_2 allyl), 5.97-6.08 (2H, -CH allyl), 6.89-6.93 (4H, -CH aromatic), 7.93-8.02 (4H, -CH aromatic).

Step 4: Preparation of Isosorbide 2,5-bis(4-Glycidyloxybenzoate)

In a dry 3-neck Morton round bottom flask, equipped with a Teflon paddle stirrer, a condenser, and a nitrogen line. 221.4 g (0.476 mol) of isosorbide 2,5-bis(4-allyloxybenzoae) and 600 ml of DCM were charged and stirred at 0-5°C. 220g (0.95 mole) of 75% 3-chloroperbenzoic acid in 600 ml DCM were charged to the reaction mixture over one hour, and the reaction was run for up to 48 hours in room temperature. Additional charges of 3-chroloperbezoic acid might be needed to complete the epoxidation. The precipitated 3-chlorobenzoic acid was then filtered off and the reaction product was mixed with 100 g of potassium carbonate to remove any remaining acid. The remaining precipitates were filtered, and the reaction product was mixed with anhydrous magnesium sulfate to remove any remaining water. DCM was removed by rotary evaporator and the off-white solid product was recrystallized with 300 ml ethyl acetate (60% yield and >98%, mp 112-113°C).
^{1}H NMR ($CDCl_3$- δ, ppm): 2.78 (2H, -CH oxirane), 2.93 (2H, -CH oxirane 3.37 (2H, -CH oxirane), 3.9-4.1 (6H, 4H -CH_2 isosorbide, 2H-CH_2 glycidyloxy), 4.29-4.31 (2H, -CH_2 glycidyloxy), 4.67 (1H, -CH isosorbide), 5.03 (1H, -CH isosorbide), 5.39 (1H,-CH isosorbide), 5.46 (1H-CH isosorbide) , 6.94 (4H, -CH aromatic), 7.9 (2H, -CH aromatic), 8.0 (2H, -CH aromatic).

2. Synthesis of ISB3GBMA Intermediates

The synthesis of the ISB3GBMA intermediates parallel those of ISB4GBMA intermediates with the following differences:

(1) Allyl 3-benzoic acid : mp 74-75°C
(2) Isosorbide 2,5-bis(3-allyloxybenzoic acid) : a liquid purified by column chromatography (ethyl acetate: hexanes, 1:1 w/w)
(3) Isosorbide 2,5-bis(3-glycidyloxybenzoate): a paste purified by column chromatography (ethyl acetate:hexanes, 2:1 w/w)

3. Synthesis of ISB2GBMA Intermediates

The synthesis of the ISB2GBMA intermediates parallel those of ISB4GBMA intermediates with the following differences:

(1) Allyl 2-benzoic acid : mp 58-59°C
(2) Isosorbide 2,5-bis(2-allyloxybenzoic acid) : a liquid purified by column chromatography (ethyl acetate: hexanes, 1:1 w/w)
(3) Isosorbide 2,5-bis(2-glycidyloxybenzoate): a paste purified by column chromatography (ethyl acetate:hexanes, 2:1 w/w)

4. Synthesis of ISDGMA Intermediates

Step 1: Preparation of Isosorbide Diallyl Ether

In a three-neck round bottom flask equipped with a Teflon paddle stirrer, a condenser, a thermocouple, and a heating mantle. 73 g (0.5 mol) of isosorbide, 67 g potassium hydroxide (1.2 mol) and 67 ml DI water were charged and heated to 65°C. 173 ml (2.0 mol) of allyl bromide were added dropwise over one hour and the reaction was run for 24 hours. The reaction mixture was neutralized with few milliliters of 12N hydcrochloric acid, extracted with 120 ml of DCM, and washed with saturated sodium bicarbonate solution and water. Then mixed with anhydrous magnesium sulfate and filtered. The final

product was distilled under vacuum to afford a clear liquid of isosorbide diallyl ether (80% yield and >98% pure).

^{1}H NMR ($CDCl_3$- δ, ppm): 3.6 (1H, -CH isosorbide), 3.86-4.17 (9H, 5H -CH isosorbide, 2-CH_2), 4.46 (1H, -CH isosorbide), 4.58 91H, -CH isosorbide), 5.13-5.17 (2H – CH allyl), 5.2-5.27 (2H, -CH allyl), 5.8-5.9 (2H, -CH allyl).

Step 2: Preparation of Isosorbide Diglycidyl Ether

In a 3-neck round bottom flask equipped with a Teflon paddle stirrer, a condenser, and a nitrogen line. 56.6 (0.25 mol) of isosorbide 2,5-bis(diallyl ether) were mixed with 400 ml of DCM and stirred at 0-5°C. 138 g (0.60 mol) of 75% 3-chloroperbenzoic acid were mixed with 300 ml of DCM and charged dropwise over one hour. The reaction was run for 48 hours under room temperature. Additional charges of 3-chloroperbenzoic acid might be needed to complete the epoxidation. The precipitated 3-chlorobenzoic acid is filtered and 50g potassium carbonate were added to remove any remining acid. The mixture is filtered and the remining product is concentrated using the rotary evaporator and purified in column chromatography (ethyl acetate:hexanes 80:20) to afford a clear liquid (73% yield and >98% pure).

^{1}H NMR ($CDCl_3$-δ, ppm): 2.51-2.57 (2H, -CH oxirane), 2.71-2.75 (2H, -CH oxirane), 3.06-3.12 (2H, -CH oxirane), 3.29-4.07 (10H, 6H -CH isosorbide, 4H - 2-CH_2), 4.43-4.47 (1H, -CH isosorbide), 4.57-4.63 (1H, -CH isosorbide).

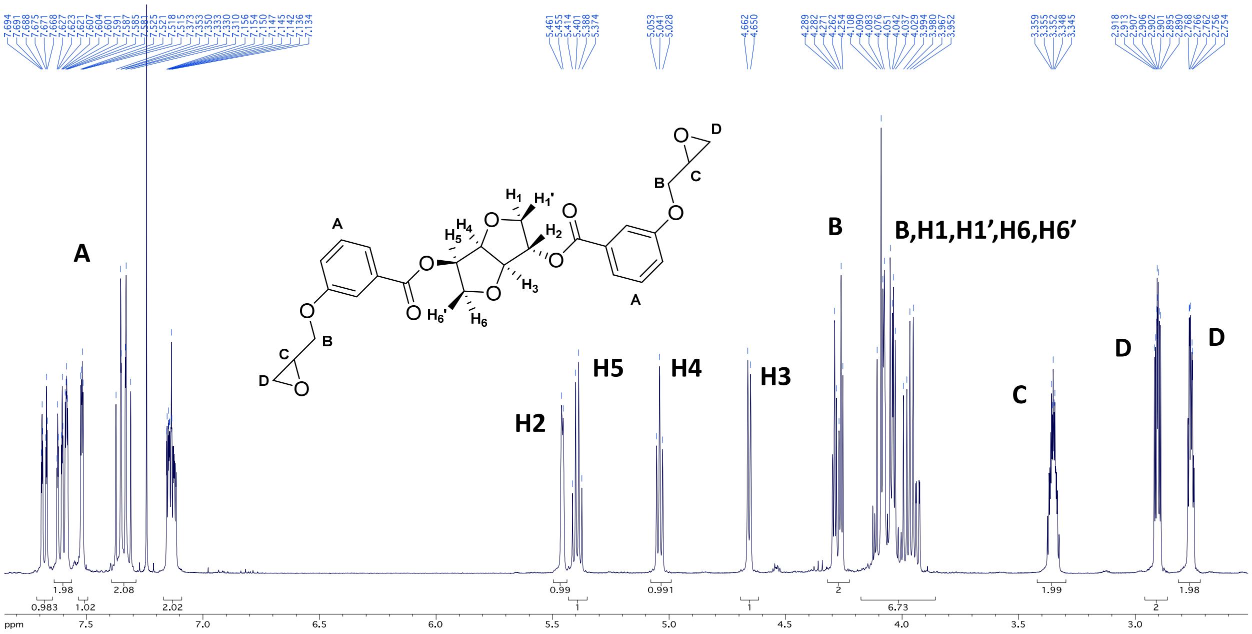

Figure S1. H NMR spectrum of Isosorbide 2,5-bis(3-glycidyloxybenzoate).

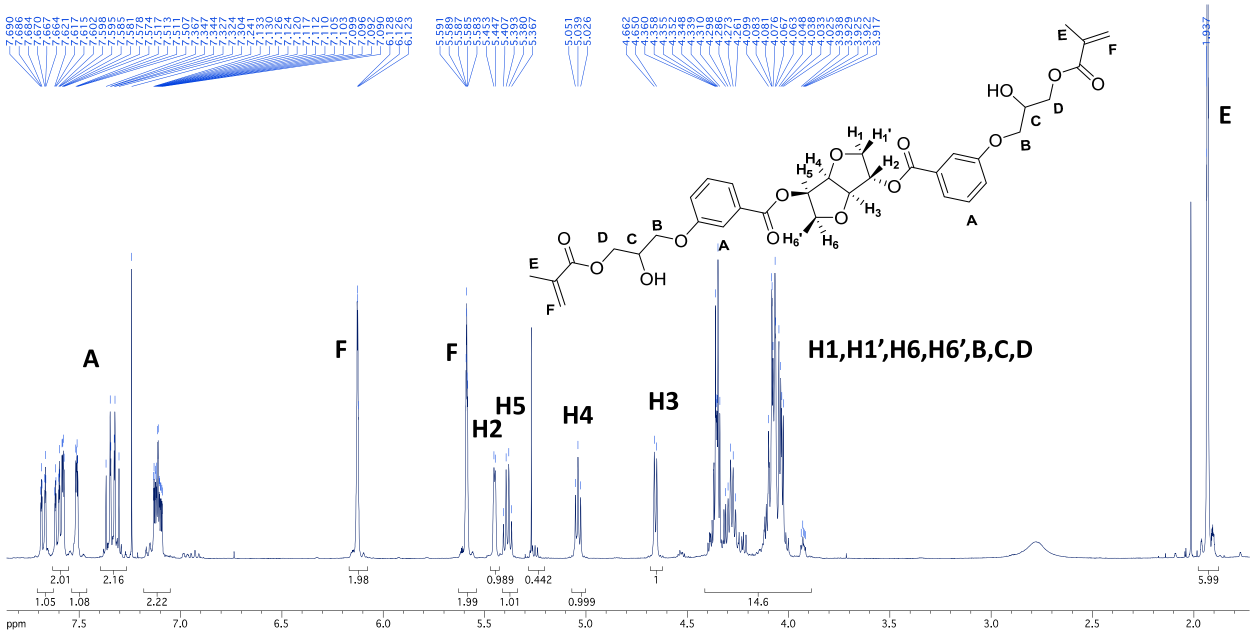

Figure S2. H NMR spectrum of Isosorbide 2,5-bis(3-glyceryloxybenzoate) dimethacrylate.

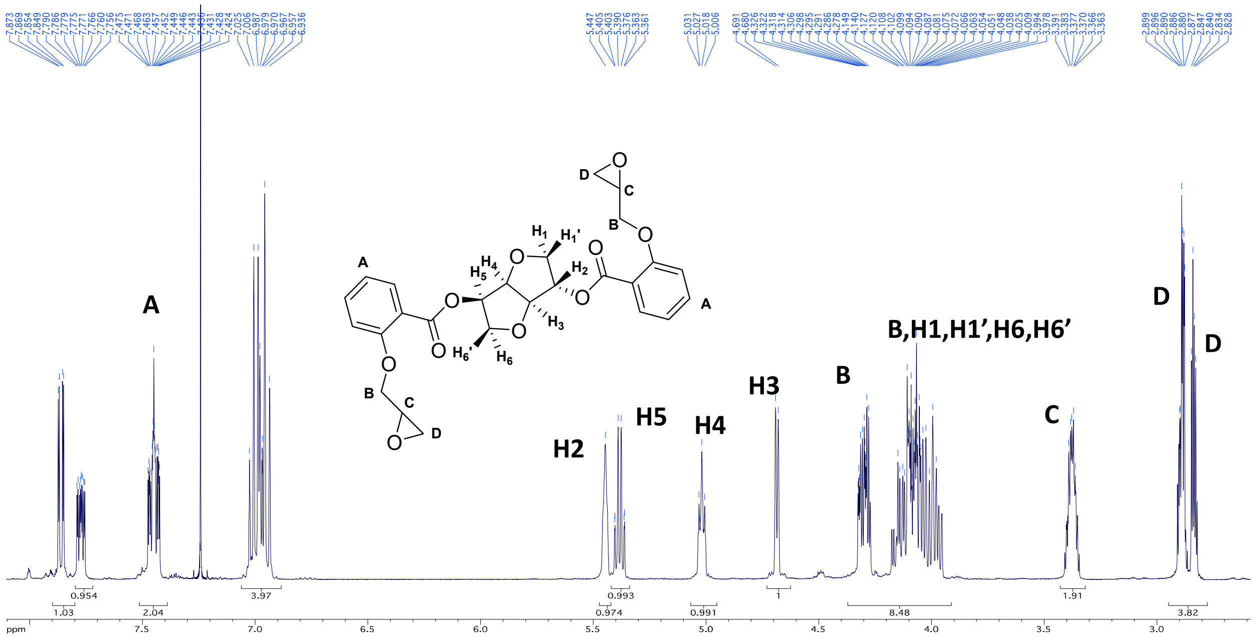

Figure S3. H NMR spectrum of Isosorbide 2,5-bis(2-glycidyloxybenzoate).

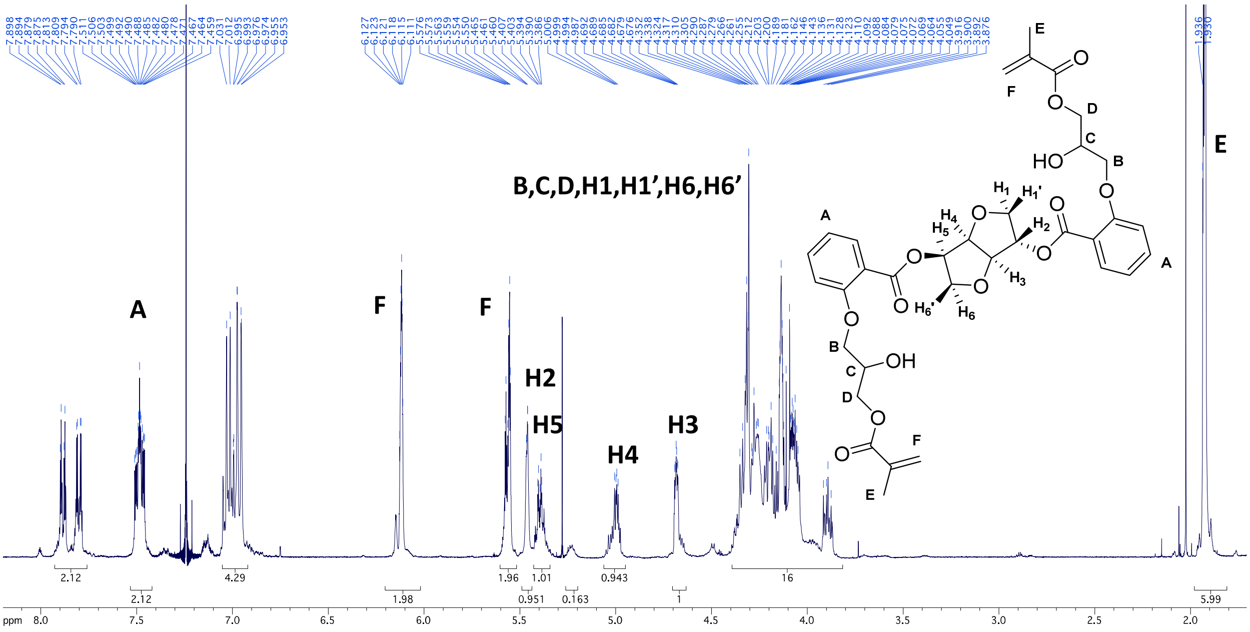

Figure S4. H NMR spectrum of Isosorbide 2,5-bis(2-glyceryloxybenzoate) dimethacrylate.

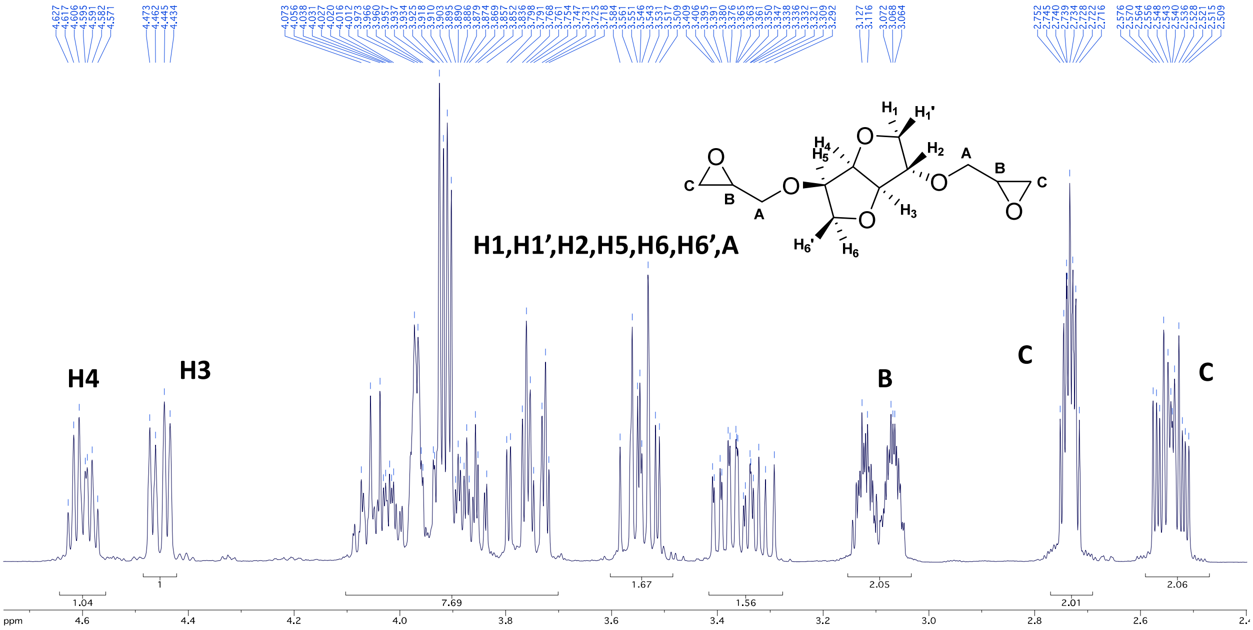

Figure S5. H NMR spectrum of Isosorbide 2,5-bis(glycidylether).

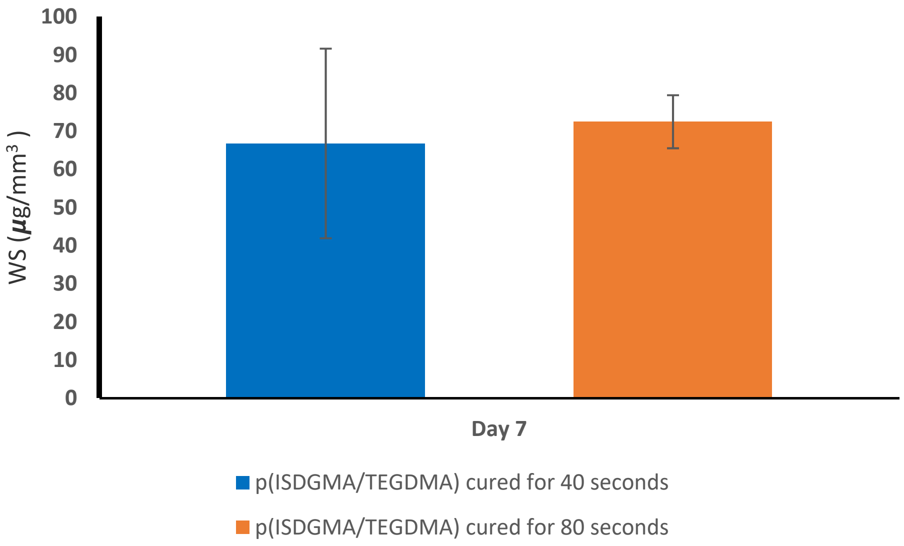

Figure S6. Water sorption of p(ISDGMA/TEGMDA) when cured at different time lengths.
